# Ensemble Learning Using Fuzzy Weights to Improve Learning Style Identification for Adapted Instructional Routines

**DOI:** 10.3390/e22070735

**Published:** 2020-07-02

**Authors:** Christos Troussas, Akrivi Krouska, Cleo Sgouropoulou, Ioannis Voyiatzis

**Affiliations:** Department of Informatics and Computer Engineering, University of West Attica, 12243 Egaleo, Greece; akrouska@uniwa.gr (A.K.); csgouro@uniwa.gr (C.S.); voyageri@uniwa.gr (I.V.)

**Keywords:** adaptive instructional routines, automatic detection of learning style, ensemble learning, fuzzy weights, mobile learning

## Abstract

Mobile personalized learning can be achieved by the identification of students’ learning styles; however, this happens with the completion of large questionnaires. This task has been reported as tedious and time-consuming, causing random selection of the questionnaires’ choices, and thus, erroneous adaptation to students’ needs, endangering knowledge acquisition. Moreover, mobile environments render the selection of questionnaires’ choices impractical due to confined mobile user interfaces. In view of the above, this paper presents Learnglish, a fully developed mobile language learning system incorporating automatic identification of students’ learning styles according to the Felder-Silverman model (FSLSM) using ensemble classification. In particular, three classifiers, namely SVM, NB and KNN, are combined based on the majority voting rule. The major innovation of this task, apart from the ensemble classification and the mobile learning environment, is that Learnglish takes as input a minimum number of personal (i.e., age and gender) and cognitive characteristics (i.e., prior academic performance categorized using fuzzy weights), and solely four questions pertaining to the FSLSM dimensions, to identify the learning style. Furthermore, Learnglish incorporates adapted instructional routines to create an individualized learning environment based on students’ learning preferences as determined by their style. Learnglish was fully evaluated with very encouraging results.

## 1. Introduction

During the last years, the rapid development of the internet and communications technology has provoked remarkable changes to many aspects of people’s everyday life. Especially in the field of education, people of all ages use technology to support their learning and knowledge acquisition through e-learning systems [1]. As such, the ever-increasing need for digital education, overcoming the barriers posed by place and time, has led to mobile learning (m-learning), which is learning across multiple contexts using personal electronic devices. To this direction, mobile technology provided all indispensable tools to support m-learning either in the context of formal or informal education. When m-learning is used for the tutoring of foreign language, typically called as second language, a new research area is born, named mobile-assisted language learning (MALL). MALL involves the support of students’ language learning with the increased use of mobile technologies and handheld devices. However, digital learning software and specifically MALL applications are oriented to a heterogeneous group of learners having different knowledge background, preferences, needs and interests [2]. Therefore, the emerging challenge is to develop learning technology systems that will be enriched with intelligent techniques in order to offer dynamic adaptation to students’ personal traits (such as knowledge, needs and preferences) and create a personalized learning experience.

A solution to this challenge is the employment of personalization techniques [3]. Personalization techniques are responsible to identify the students’ needs and preferences and respond to them by building an individualized environment. They also assist the learning technology systems respond to students’ personal needs by adapting the learning material to them in a dynamic way [4]. As such, students can receive specific domain knowledge to be taught, being adapted to their model and helping them decreasing the learning time. Furthermore, personalization techniques serve for the detection and utilization of students’ learning styles [5]. The idea of individualized learning styles influences education aiming to account for differences in individuals’ learning. Learning styles detection underlines the fact that all people can be classified according to their “style” of learning, since people differ in how they learn.

In view of the above, this paper seeks to answer three research questions:

(1) Can the learning style be identified automatically using only a minimum number of student characteristics? (RQ1)
(2) Can m-learning environments be adjusted properly to students’ needs and preferences providing individualized tutoring? (RQ2)
(3) Do mobile applications provide a more flexible, attractive and effective learning environment against web ones? (RQ3)

To this end, this paper presents an ensemble method for improving the automatic identification of students’ learning styles according to the Felder Silverman learning style model (FSLSM). This ensemble learning algorithm consists of three well-known classifiers, namely support vector machine (SVM), naïve Bayes (NB) and K-nearest neighbors (KNN), combined by the majority voting technique. FSLSM was chosen since it is a learning style model which is tailored to technology-aided instruction and provides a more detailed description of its dimensions in comparison to other learning styles, and thus, it is considered as one of the most effective models [6]. FSLSM distinguishes four dimensions for learning styles, namely perception, input, processing and understanding. The input of ensemble method is the minimum required, i.e., only three student characteristics based on personal and cognitive attributes; namely age, gender, prior academic performance, and only four questions pertaining to the FSLSM dimensions. In particular, the prior academic performance is determined using fuzzy weights, since there is a degree of uncertainty for the category to which a grade belongs; for instance, a grade of 75 % can be characterized as partially good and partially very good. The output of the automatic identification of students’ learning styles is the dimensions to which each learner belongs without having to answer all the questions in the FSLSM questionnaire, which is a tedious and time-consuming task. Then, based on the automatic identification of students’ learning styles, instructional routines are involved in order to provide a more personalized learning experience based on their learning needs and preferences. 

As a testbed for our research, we developed a mobile application, called Learnglish, for the tutoring of English as a foreign language oriented to Elementary school students which incorporates the aforementioned module. It is further accentuated that in mobile environments, it is even more difficult for students to answer an online questionnaire in order to find out their learning style due to the limited ability for selection and reading. Thus, automation can be seen as the only option for identifying the learning styles in such environments. Learnglish offers personalization and adaptation to students regarding their learning style and preferences, creating a learning environment which determines what a student is taught, how s/he is taught and if s/he is supported by peers in group activities. Learnglish was used by elementary school students and was fully evaluated with positive and encouraging results.

The remainder of this paper is organized as follows: In Section 2, an overview of the related scientific literature is presented. In Section 3, an in-depth analysis of the ensemble automatic learning style identification of learning styles using ensemble classification and the adapted instructional routines is presented. In Section 4, the evaluation results are discussed. Finally, in Section 5, the conclusions are drawn and the future steps are described.

## 2. Related Work

Research interest has been placed on the automatic detection of students’ learning styles as a means to create a student-centered learning experience. Towards this direction, researchers have used several methods. For example, a review paper [7] points out that Bayesian network and rule-based algorithms have been used for the automatic detection of the learning styles and that the detection of learning styles could enhance the adaptive learning content delivery. Another review article [8], authors state that most researchers use the students’ personality traits in adaptive learning environments as an input to the learning style identification techniques. Also, in [9], the authors employed a tree augmented naïve Bayes network with greater detection accuracy in comparison to the Bayesian network. Another research effort [10] suggests the employment of neural networks and fuzzy logic techniques for the learning style detection; as reported by the authors, this resulted in the upgrading of the effectiveness of e-learning and knowledge-management systems. Fuzzy logic has been used in [11] as a means to automatically detect learning styles, taking as input solely learner’s navigational accesses data with promising results for the efficiency and effectiveness of the entire learning process. As reported by other studies [12,13], the learners’ skills and their prior knowledge are the key characteristics that have been used towards the automatic detection of learning styles; both research efforts focused on mapping students’ skills in terms of knowledge of facts and meaning and integration of and application of knowledge being closely related to learning style. In [14], the authors present an automatic approach for detecting students’ learning style based on web usage mining; specifically, the students’ log files were classified using clustering algorithms with a view to detect their learning style. In [15], the authors explored four computational intelligence techniques (artificial neural network, ant colony optimization, genetic algorithm, and particle swarm optimization) to improve the accuracy of learning style detection by employing the FSLSM and the students’ behavior. In [16], the authors presented an inference engine incorporating a neural network and using certain observed parameters of learners’ behaviors to infer the learning style. In [17], the authors investigated the employment of the students’ behavior in a decision tree classifier for the detection of their learning style. Finally, in [18], the authors proposed a model based on an artificial neural network to improve the accuracy of automatic learning styles identification.

Summarizing, it needs to be noted that in all the aforementioned studies, a desktop user interface is used and researchers use intelligent techniques (mainly Bayesian neural networks, rule-based algorithms and fuzzy logic) to identify learning styles (Table 1). Furthermore, they use multiple and numerous characteristics as predictors of learning styles, such as students’ interaction styles (e.g., performance, time spent in assignments, etc.), sequencing actions, preference in assessment units, gender, prior education, culture and ethnicity, background knowledge level, level of intelligence (IQ classification), cognitive characteristics (such as learning capabilities), learning goals and motivation level. However, after a thorough investigation in the related scientific literature, we came up with the result that for the automatic identification of the learning style, the research efforts have not employed yet ensemble classifiers taking as input only a minimum number of students’ personal and cognitive characteristics, such as their age and gender and prior academic performance. There is scope for a lot of research and improvement.

## 3. Automatic Identification of Learning Styles and Instructional Routines

In the related scientific literature, there are several learning style models, providing different classification of learners, according to their preferences in the educational process. However, in this work, the Felder Silverman learning style model (FSLSM) [19] was chosen, given that it comprises of styles of a learner being presented in depth in comparison to other models distinguishing learners in fewer styles [6]. Furthermore, another reason of this selection lies in the fact that FSLSM focuses on learning inclinations; this accentuates the possibility that even though a learner demonstrates a high preference in a specific style, s/he can occasionally act, showing a learning behavior of a different learning style [6]. More specifically, according to FSLSM, learning styles classification is not strict; for example, if a student is visual, preferring images, s/he can sometimes want to see text during the educational process.

FSLSM is a model of learning styles namely of the manner according to which a student learns in a more successful way. It is based on individual characteristics and preferences and is often used in e-learning. Indeed, individual learning styles are significant to be examined for effective knowledge acquisition because different students learn in different ways. FSLSM distinguishes and classifies learners in four dimensions, which include values being associated with a binary division of learning styles and denoting the pace of the educational process. 

As mentioned above, FSLSM has four dimensions and each dimension specifies two contrary learning styles as follows:

- Input: This dimension identifies how students prefer to receive external information or how to be presented external stimuli. The learning styles of the input dimension are:
  ○ Visual: A visual learner prefers to see pictures, graphs, charts, diagrams and any kind of information that is easily perceived simply by looking at it. Visual learners can better recall such kind of information.
  ○ Verbal: A verbal learner prefers to listen to discussions and explanations, thriving when a mix of hearing and speaking is involved. As such, they tend to remember better what they hear.
- Processing: This dimension determines how students prefer to process information as well as how this information is transformed to knowledge. The learning styles of Processing dimension are:
  ○ Active: An active learner prefers activities which demand active participation or experimentation. As such, active learners are keen on collaborating with peers or being part of a group.
  ○ Reflective: A reflective learner prefers working alone. They avoid collaborative activities or group exercises since they tend to think a lot about information being delivered to them.
- Perception: This dimension identifies the type of information a student prefers to perceive. The learning styles of the Perception dimension are:
  ○ Sensing: A sensing learner prefers activities like problem-solving, experimenting, and generally they are practical focusing on facts and procedures. Such learners are good memorizers and careful but slow while learning.
  ○ Intuitive: An intuitive learner prefers dealing with theories and meanings. They are keen on tackling with new ideas, innovative tasks, and working with symbols. Such learners can be quick in complicated issues but they are careless.
- Understanding: This dimension describes the way with which learners prefer to organize and progress towards understanding information. The learning styles of the Understanding dimension are:
  ○ Sequential: A sequential learner prefers a logically ordered progression when dealing with problem solving. A linear learning progress and learning in small incremental steps can help them work with new concepts only if they have understood it, even partially.
  ○ Global: A global learner prefers to have a holistic view of the new information and to take large steps forward. Global learners present an instinctive pace in learning and have difficulty in conceiving fragmentary information and explaining results in knowledge acquisition.

In [20], an index of learning styles is presented; it constitutes a questionnaire with 44 questions, being divided in the four dimensions (11 questions in each dimension). The students’ learning preferences are identified by the answers in each question, which has two mutually exclusive values.

However, in the related scientific literature [9,21,22,23,24,25,26,27], a lack of students’ motivation to use a questionnaire in order to determine their learning preferences is observed due to the following reasons. Firstly, students consider that filling out the questionnaire is a boring task requiring an additional load of work. Also, even if students provide their answers to the questionnaire, they can be arbitrarily chosen. Moreover, the wording and formulation of the questionnaire may confuse and affect students, causing a selection of answers which are regarded as more suitable and possible. Furthermore, learning styles can change over time; hence, the results of a static questionnaire can be seen as obsolete as a learner progresses. On the other hand, automatic mechanisms for learning styles detection can be more accurate and error-free [25].

In view of the above, Learnglish employs a module for the automatic identification of the learning styles (AILS), based on FSLSM, using only a few attributes. AILS uses ensemble classification analysis, and specifically the SVM, NB and KNN classifiers combined by majority voting rule, in order to classify students into the proper learning style. Firstly, the ensemble learning algorithm is trained based on a set of data, and afterwards, the class of the instance is predicted considering the highest number of votes it takes among the base classifiers. 

**Figure 1:**
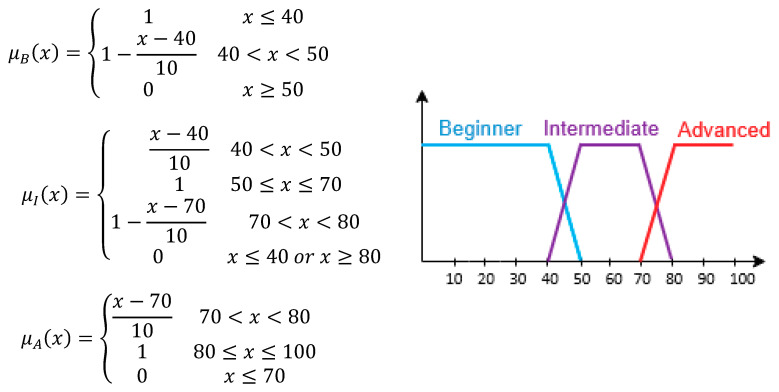
Fuzzy weights of students’ prior academic performance.

Towards identifying the students’ learning style, Learnglish takes as input specific students’ characteristics which are the following:

- Age: The age is a very important characteristic that can affect the learning styles of the students as reported in [28,29,30,31,32,33]. For example, students of younger ages have a stronger preference for visualized objects than older students. On the other hand, older learners prefer focusing on details and experience competitive activities as well as listening and reading activities. Also, younger students prefer collaborating with peers, creating lists of objects and generally direct communication. It is inferred that learning styles can be altered as a person gets older. As such, Learnglish considers the learning style as a dynamic characteristic that can change when several students’ characteristics, like age, are modified. The age is asked to be provided at the first interaction of the learner with Learnglish. This characteristic can take values for four age categories, namely prepubescent (6–12 years old), younger (13–18 years old), average (19–45 years old) and older learners (more than 46 years old).
- Gender: The gender is also a very significant characteristic that can influence the identification of learning styles, as reported in [28,29,30,31,33]. According to the aforementioned studies, tailored teaching strategies, based on learning styles, can be offered to female and male learners. Knowledge acquisition and skill refinement can be enhanced by the identification of student’s learning style, as altered by their gender. For example, male learners prefer auditory and reading in contrast to female learners who prefer more composition activities. The gender is asked to be provided at the first interaction of the learner with Learnglish. This characteristic can take two values: female and male.
- Prior academic performance: Prior academic performance constitutes an important factor for the identification of learning styles, as reported in [30,33]. There is a connection between the learning style and preferred teaching strategies with the students’ prior academic performance. For example, learners with a high knowledge level prefer more complex knowledge delivery activities, such as diagrams or mathematical equations, whereas learners with a lower knowledge level prefer examples and images. The prior academic performance is computed at the first interaction of the learner with Learnglish, when s/he asked to answer in preliminary questions pertaining to the English language domain knowledge. The determination of the prior academic performance of students is not a simple procedure; on the contrary, it is a process full of uncertainty. For example, if the student’s academic performance is 70/100, it cannot be simply characterized neither as good nor as advanced. Both characterizations can be considered with a degree of accuracy. Fuzzy logic can be used as the key for this situation. For our case, the academic performance was depicted by the use of three fuzzy weights, which are: Beginner (B), Intermediate (I), Advanced (A). Their membership functions (x is the score of the student) are as shown in Figure 1:

The above membership functions determine the values of the three fuzzy sets (μ_B_, μ_I_, μ_A_) which in turn depict the prior academic performance of each student (Figure 1). These values can span from 0 to 1. In more detail, when the value is equal to 1, then it means that the student has complete knowledge for the domain being taught. As such, the sum of the partition value of each fuzzy set representing the academic performance for a domain concept each time is equal to 1. Hence, the equation μ_B_(x) + μ_I_(x) + μ_A_(x) = 1 stands. The aforementioned fuzzy sets, as well as the thresholds of their membership functions, have been specified by 16 experts of different domains: 10 of them are computer science experts serving as faculty members in computer science departments in public universities, while 6 of them are pedagogical experts, serving as faculty members in education departments in public universities as well. All these experts have great experience (more than 10 years) in teaching approaches, adaptive instruction to students’ academic needs and learning design and assessment. More specifically, they were asked to determine the different levels of a student’s progress and performance descriptively, as well as the intervals of a student’s degree of success, typifying each of these knowledge levels.

- Four questions pertaining to the FSLSM dimensions: Towards a successful identification of students’ learning styles, a group of four questions have been delivered to students to answer them before their first interaction with Learnglish. Each one of these questions represents one of the four dimensions of FSLSM, namely Understanding, Perception, Input and Processing. The four questions are the most representative of each dimension and their selection is based on [6]. The four questions being related to the FSLSM dimensions are as follows:
  ○ “When I study a new topic, I prefer (a) to learn the domain being taught in clear steps and remain focused. (b) have a general picture of the links/relationships between that topic and related topics.” This question is related to the Understanding dimension.
  ○ “I prefer lessons that accentuate (a) specific material (data or facts). (b) theoretical and conceptual material (theories, concepts).” This question is related to the Perception dimension.
  ○ “When I am given data and information, I prefer to (a) concentrate on the figures, charts and graphs gingerly. (b) put my attention to oral information or written text, tabulating the results.” This question is related to the Input dimension.
  ○ “I prefer to learn (a) in a study group. (b) alone.” This question is related to the Processing dimension.

### 3.1. Ensemble Learning for Improving Automatic Learning Style Identification

**Figure 2:**
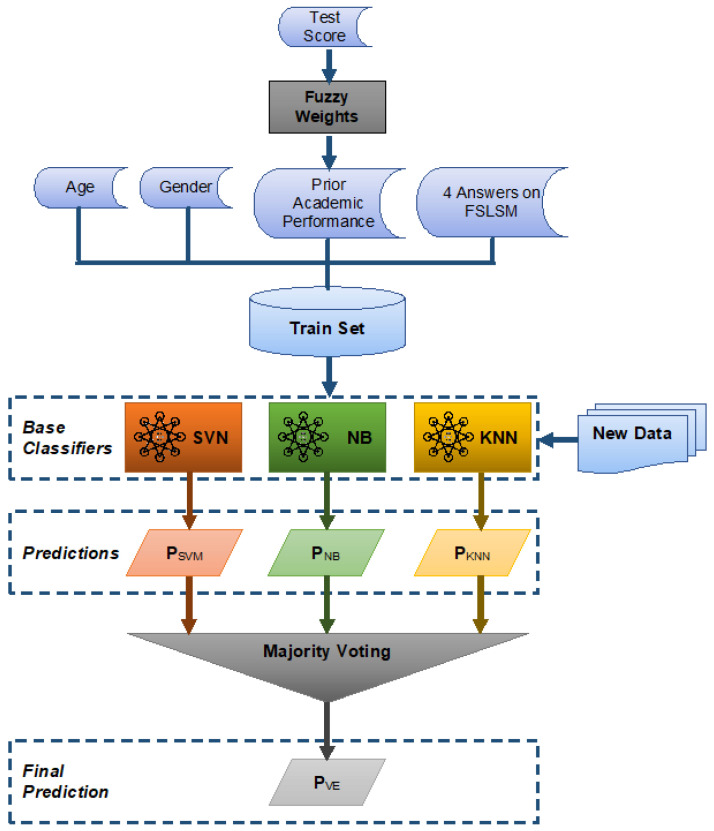
Automatic learning style identification.

The classification of students takes place using ensemble learning (Figure 2). In particular, the Voting Ensemble algorithm was chosen using three base classifiers, namely SVM, NB and KNN, which are trained and valuated in parallel and afterwards, their individual predictions are combined according to the majority voting rule. Thus, the final output is given by the following equation:

(1) $$P_{VE}\left(\vec{s_{i}}\right)=argmax\;\left(P_{SVM}\left(\vec{s_{i}}\right),P_{NB}\left(\vec{s_{i}}\right),P_{KNN}\left(\vec{s_{i}}\right)\right)$$

where $\vec{s_{i}}$ is the input data of student i, and $P_{SVM}$, $P_{NB}$, $P_{KNN}$ are the predictions of the base classifiers, while $P_{VE}$ is the final prediction of voting ensemble algorithm. The argmax function is the majority voting rule used, which returns the class with the largest predicted probability.

These base models were selected based on the testing of a set of five classifiers including Multilayer Perceptron (MLP), SVM, NB, KNN and Decision Tree (C5) on a sample of the whole dataset. After combinations of the algorithms, the chosen algorithms were reported to have a higher performance in terms of execution time and accuracy. 

The voting ensemble algorithm was run using the default settings for the base classifiers. Especially for KNN, the k parameter took the value 7, emerged from the formula k = √n/2 − 1 = √306/2 − 1 = 7, where n is the number of the sample (n = 306 students) [34]. The whole process explaining the operation of the ensemble algorithm used in Learnglish can be shown by the following pseudocode:
**Algorithms 1** Input:    SC: Struct {Age, Gender, PAP, A1, A2, A3, A4, LS}//Node of student characteristicsT[]: SC    //Data in Training setBST[]: SC     // Bootstrap sample of T      BC[]: Classifier   //Array of base classifiers      t: SC       //instance, in the form of vector, for classificationOutput: C*: {GIVeA | GIVeR | GIViA | GIViR | GSnVeA | GSnVeR | GSnViA | GSnViR | SqIVeA | SqIVeR | SqIViA | SqIViR | SqSnVeA | SqSnVeR | SqSnViA | SqSnViR} //Class in which t will be classified     C[]: C* //Class in which t will be classified by each base classifierStart_AlgorithmBC = {SVM, NB, KNN}For i = 0 to BC.length – 1 do  BST = Bootstrap(T)  //generate a bootstrap sample from T  BC[i].Train(BST)   //train the base classifier from the bootstrap sample  C[i] = BC[i].Predict(t) //predict the class for the instance tEnd For  C* = argmax (C.Values())End_Algorithm

Table 2 presents an example of operation for the automatic identification of learning styles using the ensemble algorithm. In this example, 306 students have taken place and have provided the information to Learnglish (so that their vector is created). Then, the ensemble algorithm is asked to classify a new student, named Nadia. Towards this direction, the ensemble algorithm searches for the class that will receive the highest number of votes. Running the algorithm for the new student, we take as a result that classifiers 1 and 2 give as result the class 0 (meaning “Global-Intuitive-Visual-Active”) while classifier 3 gives as result the class 1 (meaning “Global-Intuitive-Verbal-Active”), as shown in the following formula:

(2) $$P_{VE}\left(\vec{s_{i}}\right)=argmax\;\left(P_{SVM}\left(\vec{s_{i}}\right),P_{NB}\left(\vec{s_{i}}\right),P_{KNN}\left(\vec{s_{i}}\right)\right)=argmax\;\left(0,\;0,\;1\right)=0$$

Therefore, the learning style that is assigned to Nadia is the following: Global-Intuitive-Visual-Active. 

### 3.2. Instructional Routines of Learnglish for Developing an Individualized Learning Environment

After the learning style is automatically identified, Learnglish uses a corresponding instructional routine in order to personalize the educational experience to students based on their learning style. Based on FSLSM, each student is assigned one dimension from the four categories (Understanding, Perception, Processing and Input), and thus, a different routine is used for each one of the 2^n^ = 16 learning style options (n = the values of the four dimensions and the formula 2^n^ comes from the mathematical combinatorics). Table 3 illustrates the instructional routines that are developed so that Learnglish can adapt its content to the students confronting the specific needs and preferences of them. For each one of the 16 learning style classifications, an aggregated routine is delivered based on each one of the four dimensions. For example, if a student is global, intuitive, verbal and active, the following adapted routine is delivered: The learning material is open and students are given advice to move from chapter to chapter based on their learning needs and the exercises involve evaluation of holistic thinking. The learning material includes a lot of theoretical concepts without factual examples. The learning material includes written text and a possibility of listening to the theory by a speaking agent. Learnglish proposes study groups where students can talk about the learning material and also it creates student groups who are given a group assignment to solve it with peers.

## 4. Evaluation Results and Discussion

This section may be divided by subheadings. It should provide a concise and precise description of the experimental results, their interpretation as well as the experimental conclusions that can be drawn.

The evaluation process is essential for the life cycle of software to ensure the accomplishment of system’s goals, analyze its effectiveness, and identify areas for improvement. To this direction, a multi-dimensional evaluation is conducted, including the following:

- Evaluation of the proposed ensemble learning algorithm for the automatic identification of learning style using confusion matrix, answering RQ1.
- Evaluation of the quality and functionalities of the developed mobile system (Learnglish) using the multidisciplinary framework by [35], answering RQ2.
- Comparison of the presented mobile application (Learnglish) with a corresponding web one applying statistical hypothesis tests (t-test), answering RQ3.

**Figure 3:**
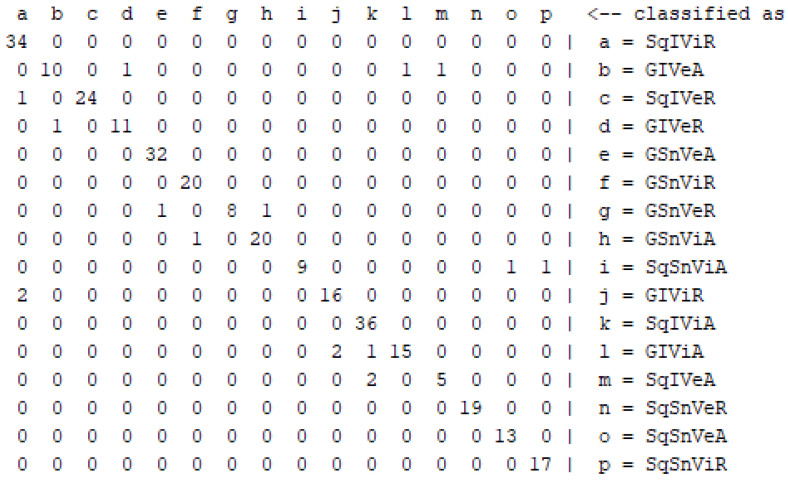
Confusion matrix for ensemble learning algorithm.

Regarding the evaluation of the proposed voting ensemble algorithm, a questionnaire including the students’ age and gender, the four questions corresponding to FSLSM dimensions and the complete Index of Learning Styles questionnaire (based on FSLSM) with the 44 questions is delivered to 306 students of fifth and sixth grade classes of three elementary schools (consisting of two classes per grade with around 25 students each one). Moreover, students gave the preliminary test used in our developed mobile system in order their prior academic performance to be computed. The scores of this test were processed using fuzzy weights in order the student knowledge level to be defined efficiently. All the above are required for training the algorithm properly and evaluating its performance. Therefore, the students’ characteristics (age, gender, prior academic performance) and the answers of the four questions are used as input to our algorithm to identify their learning style, whereas the result of the complete Index of Learning Styles questionnaire [20] determines the correct student learning style. Our approach led to achieving 94.44% accuracy, 0.945 precision and 0.944 recall, whereas the simple classifiers reached lower performance. In particular, SVM achieved 89.54% accuracy, 0.898 precision and 0.895 recall, NB had 88.24% accuracy, 0.886 precision and 0.882 recall, and KNN reached 84.97% accuracy, 0.863 precision and 0.850 recall. Table 4 and Table 5 illustrate the voting ensemble algorithm results in comparison with the simple classifiers regarding the different metrics, whereas Figure 3 depicts the confusion matrix of the ensemble learning. The results of voting ensemble algorithm evaluation were very encouraging, showing that the proposed algorithm outperforms the simple classifiers, and furthermore in relation to the attributes used for the learning style classification identifies efficiently the students’ learning style with the less student effort in terms of data entry (RQ1).

Evaluating the developed mobile application, 56 students from a public elementary school participated. The students were from two different classes of the same school (28 students per class). Also, the students were of the same grade (sixth grade of elementary school). The first class (Class A) evaluated Learnglish through smartphones with which all students were provided for the sake of the experiment. The second class (Class B) evaluated the educational system through its conventional web interface that functions through personal computers. This segmentation was very significant in order to measure the mobile platforms effectiveness against the conventional way of tutoring through computers and on the other hand to measure the degree of personalization to students’ needs and preferences. Consequently, both classes had given the right equipment (such as hardware) and then they received a detailed demonstration of the utilization of both educational platforms. Each class used the corresponding application for a month and thus they had plenty of time to spend interacting with the systems. After the completion of their interaction (Class A with mobile devices and Class B with personal computers), all students were given questionnaires to complete with guidance from the evaluators and also their four teachers. It needs to be noted that school teachers also provided very valuable help in the whole evaluation study since they also participated both in the presentation of the ITS to the students and also provided assistance to their students while they interacted with the educational platform. Moreover, it was observed that students became familiar easily and very quickly with the educational software, its features and its functionalities. Their interest was undiminished during the whole period of their interaction with the educational application. 

Regarding the quality and functionality of our mobile system used by Class A, a multidisciplinary framework, presented by [35], was applied. This framework was further examined and evaluated in [36,37] in order to attest its quality. The framework is comprised of the following metrics:

- Usability: Usability is one important factor of the overall acceptability of the system. It comprises of aspects pertaining to the user interface, such as easy and efficient in use, easy to remember, having few errors and being pleasing.
- Pedagogical usability: Pedagogical usability involves the pedagogical affordances of the system, namely the support for development of learning skills, learning and tutoring process and organization. More specifically, it concerns the pedagogical design of the learning technology system and focuses on the tools, content, interface and tasks supporting learning and tutoring process. Pedagogical objectives, including learning objectives, are measured in this dimension as an important factor of the organization of the teaching process, the development of the learners’ skills, the development of the quality of teaching and the testing methods of learners.
- Accessibility: This metric concerns the design of the accessibility using established guidelines and checklists to evaluate the accessibility of web-pages.
- Informational Quality: This metric concerns the high quality of the informational content in terms of accuracy, authority, objectivity, currency and coverage. This metric also includes the presentation of information and content from a teaching perspective.

The evaluation study was held with the use of self-supplemented scale questionnaires with closed questions delivered to the students. Based on the aforementioned evaluation framework, 43 questions, related to the framework’s metrics, have been delivered to students and are as follows:

- Eleven questions regarding the Usability metric
- Eighteen questions regarding the Pedagogical Usability metric
- Eight questions regarding the Accessibility metric
- Six questions regarding the Informational Quality metric.

Table 6 summarizes the questions that were delivered to students. For the questions, the evaluators used a scale from 1–10, where 1 is the lowest grade and 10 is the highest grade. The validity and reliability of the questionnaire have been explored by its creators [35,36,37].

**Figure 4:**
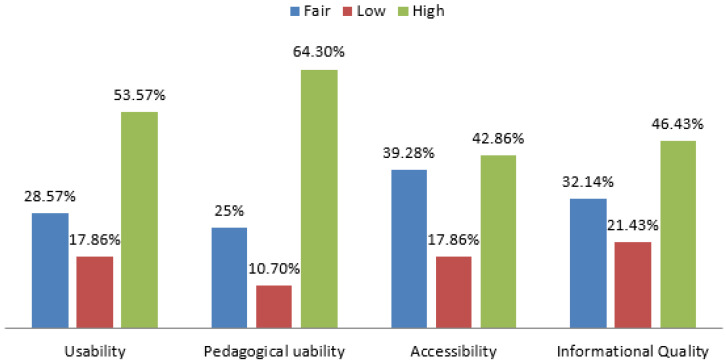
Evaluation results based on the framework metrics of [36].

Analyzing the evaluation results illustrated in Figure 4, the RQ2 is answered confirming the pedagogical affordance of adaptivity and functionality of the developed mobile system. In particular, it should be accentuated that user interface is reported to be easy and efficient during the students’ interaction with it. The user interface is involved in the metric of system’s usability. Indeed, a user interface is crucial for a tutoring system since it integrates three types of information that are needed in carrying out a dialogue: knowledge about patterns of interpretation (to understand a speaker) and action (to generate utterances) within dialogues; domain knowledge needed for communicating content; and knowledge needed for communicating intent. Furthermore, an important dimension of the metric of accessibility is the accessibility of multimedia. Based on the results, multimedia is reported to be very easily accessible in our system. This is very important considering that the system uses mobile technology and students can have access to multimedia using smartphones. Students, also, reported that the teaching strategy is well-organized. As this constitutes a dimension of the Pedagogical usability metric, it is really noteworthy that our system can have a positive impact on learning. The automatic identification of the students’ learning style along with the domain knowledge adaptation have been seen as potentially valuable tools by the learners who reported that they assisted them successfully advance their knowledge level. Finally, the presentation of the information, as a dimension of the Information Quality metric, is related to the quantity and degree of information that a student receives. This dimension is found to be highly graded; this was expected since the domain knowledge is delivered to each student based on his/her learning needs and preferences while all other kind of information is delivered to students based on their importance and relevance (e.g., statistics, etc.).

Finally, for assessing the technological affordance of the mobile technology in elementary schools and answering RQ3, the t-test (statistical hypothesis test) was employed. Hence, the results of Class A were compared to the results emerged from the evaluation of Class B. Table 7 illustrates the statistical significance of questions 1 (Rate the user interface in terms of easiness and efficiency.), 13 (Rate the overall learning process you’re your experience.) and 16 (Would you like to use this platform in your school?). For the Null hypothesis: “There is no difference between the two groups of students” the *t*-Test rejects the hypothesis for the aforementioned questions, while the remaining questions seem not to have statistical differences between the two groups of students. Since the alpha value of *t*-Test was set at 0.05 and the *p*-value is less than 0.05, there is a statistically significant difference between the means of the two trials in all questions. It needs to be noted that for Question 1, the *p*-value is 0.001756, for question 13, the p-value is 0.006476 and for Question 16, the *p*-value is 0.000199.

Based on the t-test results, it needs to be underlined that the easiness of the user interface (Question 1) is greater in the mobile application in comparison to the web application. This result was expected given that young people are very accustomed to smartphones and as such mobile user interfaces are very friendly to them. Concerning Question 13, the students’ learning experience using the mobile application surpassed their experience in using the web application. Indeed, the utilization of smartphones by young people is daily and they invest a great deal of time on them. As such, they were more focused on learning through smartphones and as a result they reported to have a better learning experience. Finally, regarding Question 16, students mentioned that they wanted to use smartphones in the elementary school in a higher degree than using a web application. This result was also expected since smartphones can give an air of entertainment to learning, thereby upgrading their knowledge level and achieving their learning goals. The students’ overall learning experience as well as their wish to use Learnglish in their school involve the fact that they approved the learning style which Learnglish detected for each one, enjoyed the delivered learning material tailored to their needs and preferences and accepted willingly the recommendation for peer collaboration.

In view of the above, it was rather expected that younger learners, who are keen on new technology would embrace learning though smartphones in school environments. Especially, when m-learning employs sophisticated techniques to become more individualized and adaptive, it is welcome by students to support their learning. The findings of this evaluation study create a fertile ground towards adopting the increasingly growing research area of mobile technology in all grades of formal education and lifelong learning. The findings, also, accentuate the fact that the employment of intelligent techniques in m-learning environments can benefit the traditional education.

Analyzing the related scientific literature, the best model built by [18] achieved 85% accuracy while the model of [15] achieved 80% accuracy. Our results (94.44% accuracy) are better than the results presented in the aforementioned research works. Our approach employing ensemble of classifiers managed to precisely and accurately detect the students’ learning styles in a higher degree.

## 5. Conclusions

To conclude, this paper presents Learnglish, which is a mobile-assisted language learning application for the tutoring of English as a foreign language. Learnglish incorporates automatic identification of students’ learning styles based on the Felder-Silverman learning style model. It takes as input personal and cognitive students’ characteristics (such as age, prior academic performance, etc.), which have been reported in the related scientific literature, as crucial factors for identifying a learning style. Then, it uses ensemble algorithms in order to reason between the appropriate style for each learner. Moreover, based on his/her learning style, Learnglish embodies routines for creating an individualized educational experience to each student based on his/her learning needs and preferences as defined by his/her learning style. 

This paper answers several research questions pertaining to the effectiveness of automatic learning styles identification using ensemble classifiers taking as input a minimum number of personal and cognitive students’ characteristics. Also, it seeks to investigate the appropriate instructional routines for adapting the learning environment to students’ learning styles and the efficiency of mobile technology supporting all students and particularly elementary school students.

The paper also presents a large-scale multi-dimensional evaluation to assess the effectiveness of the automatic learning styles identification, the quality and usability of the pedagogical affordances and the efficiency of the mobile learning environment. The evaluation results are very encouraging and promising, showing great accuracy of the proposed methods. 

Our future steps include the utilization of the presented approach in different domains and by different population in terms of its age, such as students of tertiary education. Moreover, future plans involve the creation of a hybrid learning style model, resulting from the Felder-Silverman model and other cognitive models (e.g., Gregorc Mind Style Model, Herrmann Brain Dominance instrument, etc.) in order to take into consideration different aspects that tend to affect e-learning, such as sentiments and feelings or left-brain and right-brain preferences. The identification of the hybrid learning style models will be conducted automatically using ensemble of classifiers or other intelligent techniques. Finally, given that the presented approach of the ensemble algorithm can be extended to be used in other fields as well, we tend to explore its effectiveness in predicting characteristics of products and recommend them to potential buyers in e-shops. 

## Figures and Tables

**Table 1:** Related work.

Study Reference	Characteristics	Technology	Learning Style
[7]	Learning behavior (visiting the forum, sending and receiving e-mail, watching videos, carrying out exercises, communicating etc.)	Tree augmented naïve Bayesian network	FSLSM
[8]	Learners’ interactions (content, forum, chat, etc.)	Neural networks and fuzzy logic	Honey and Mumford model
[9]	Learners’ navigational data	Fuzzy logic	Behavioral patterns
[10]	Learners’ cognitive skills	Fuzzy logic	Mental processing modeling
[11]	Prior Knowledge (knowledge of fact, knowledge of meaning, integration of knowledge, application of knowledge)	Rule-based association	VARK
[12]	Web usage	Custer analysis	FSLSM
[15]	Learners’ behavior	Artificial neural network, Genetic algorithm, Ant colony system, Particle swarm optimization	FSLSM
[16]	Learners’ behavior	Artificial neural network	Gardner’s theory
[17]	Learners’ actions	Decision trees	FSLSM
[18]	Learners’ behavior	Artificial neural network	FSLSM

**Table 2:** Example of operation.

Students	Age	Gender	Prior Academic Performance	Answers to the FSLSM Dimensions’ Questions	Result for Identifying Learning Style
George	12	Male	I	a-a-b-a	Sequential-Sensing-Verbal-Active
Nick	11	Male	I	a-a-a-b	Sequential-Sensing-Visual-Reflective
Maria	12	Female	B	a-a-a-a	Sequential-Sensing-Visual-Active
Helen	11	Female	I	b-b-a-a	Global-Intuitive-Visual-Active
John	11	Male	I	b-a-b-b	Global-Sensing-Verbal-Reflective
Sophia	11	Female	A	b-b-a-a	Global-Intuitive-Visual-Active
Elias	11	Male	B	a-b-a-b	Sequential-Intuitive-Visual-Reflective
Stella	12	Female	I	b-b-a-a	Global-Intuitive-Visual-Active
Peter	12	Male	B	b-a-b-a	Global-Sensing-Verbal-Active
Natalie	12	Female	I	b-b-b-a	Global-Intuitive-Verbal-Active
Nadia	12	Female	I	b-b-b-a	?

**Table 3:** Adapted instructional routines for each FSLSM dimension.

FSLSM Options	Learnglish Instructional Routines
Sequential	The learning material is delivered in logical and incremental steps and the assignments are separated in steps to be solved.
Global	The learning material is open and students are given advice to move from chapter to chapter based on their learning needs and the exercises involve evaluation of holistic thinking.
Sensing	The learning material includes real life examples, facts and data.
Intuitive	The learning material includes a lot of theoretical concepts without factual examples.
Verbal	The learning material includes written text and a possibility of listening to the theory by a speaking agent.
Visual	The learning material includes charts, diagrams, figures, pictures and tables.
Reflective	Learnglish proposes a topic for each learner to think on it alone and provide an answer.
Active	Learnglish proposes study groups where students can talk about the learning material and also it creates student groups who are given a group assignment to solve it with peers.

**Table 4:** Summary of classifiers’ performance evaluation.

	SVM	NB	KNN	VE
Correctly Classified Instances	274	270	260	289
	(89.54%)	(88.24%)	(84.97%)	(94.44%)
Incorrectly Classified Instances	32	36	46	17
	(10.46%)	(11.76%)	(15.03%)	(5.56%)
Kappa statistic	0.8867	0.8724	0.8368	0.9399
Mean absolute error	0.0364	0.041	0.0278	0.0069
Root mean squared error	0.1136	0.1195	0.1123	0.0833
Relative absolute error	31.46%	35.42%	24.02%	5.998%
Root relative squared error	47.22%	49.66%	46.69%	34.64%
Total Number of Instances	306	306	306	306

**Table 5:** Detailed analysis of classifiers accuracy metrics (weighted avg.).

Alg.	TP Rate	FP Rate	Precision	Recall	F-Measure	MCC	ROC Area	PRC Area
SVM	0.895	0.009	0.898	0.895	0.891	0.886	0.988	0.934
NB	0.882	0.011	0.886	0.882	0.878	0.872	0.988	0.932
KNN	0.850	0.014	0.863	0.850	0.839	0.835	0.996	0.950
VE	0.944	0.005	0.945	0.944	0.943	0.940	0.970	0.896

**Table 6:** Questionnaire [36].

Metrics		N	Questions
Usability		1	Rate the user interface in terms of easiness and efficiency.
		2	Rate the support for navigations.
		3	Rate the support for online reading.
		4	Rate the use of multimedia elements.
		5	Rate the visual design.
Accessibility		6	Rate the accessibility of multimedia.
		7	Rate the accessibility of dynamic web pages.
		8	Rate the accessibility of frames, tables, links, etc.
		9	Rate the device-independent access.
Pedagogical usability	Support for organization	10	Rate the support of educational training portal for different user groups.
		11	Rate the organization of study.
		12	Rate the organization of teaching strategies.
	Support for learning and tutoring process	13	Rate the overall learning process you’re your experience.
		14	Rate the overall teaching process.
		15	Rate the achievement of your learning goals.
		16	Would you like to use this platform in your school?
		17	Do you believe that the system identified successfully your learning style?
	Support for development of learning skills	18	Rate the degree of your self-direction.
		19	Rate the degree of interaction with peers and instructors.
		20	Rate your degree of autonomy.
		21	Did you like the way with which the system delivered the domain knowledge?
Informational Quality		22	Rate the reliability of information.
		23	Rate the presentation of information.

**Table 7:** *T*-Test: Mobile versus web application.

	Question 1		Question 13		Question 16	
	Class A	Class B	Class A	Class B	Class A	Class B
Mean	6.75	4.642857	7.821429	5.857143	8.571429	6.214286
Variance	8.712963	2.756614	8.22619	5.238095	5.291005	4.470899
Observations	28	28	28	28	28	28
Pooled variance	5.734788		6.732143		4.880952	
Hypothesized Mean Difference	0		0		0	
df	54		54		54	
t Stat	3.292299		2.832644		3.992065	
P(T <= t) one-tail	0.000878		0.003238		9.96 × 10^−5^	
t Critical one-tail	1.673565		1.673565		1.673565	
P(T <= t) two-tail	0.001756		0.006476		0.000199	
t Critical two-tail	2.004879		2.004879		2.004879	
